# A Method for Serial Tissue Processing and Parallel Analysis of Aberrant Crypt Morphology, Mucin Depletion, and Beta-Catenin Staining in an Experimental Model of Colon Carcinogenesis

**DOI:** 10.1007/s12575-010-9032-x

**Published:** 2010-05-18

**Authors:** John N McGinley, Matthew D Thompson, Henry J Thompson

**Affiliations:** 1 Cancer Prevention Laboratory, Colorado State University, 1173 Campus Delivery, Fort Collins, CO 80523, USA

**Keywords:** colon carcinogenesis, aberrant crypt foci, mucin depletion, beta-catenin, image analysis, morphometry

## Abstract

The use of architectural and morphological characteristics of cells for establishing prognostic indicators by which individual pathologies are assigned grade and stage is a well-accepted practice. Advances in automated micro- and macroscopic image acquisition and digital image analysis have created new opportunities in the field of prognostic assessment; but, one area in experimental pathology, animal models for colon cancer, has not taken advantage of these opportunities. This situation is primarily due to the methods available to evaluate the colon of the rodent for the presence of premalignant and malignant pathologies. We report a new method for the excision and processing of the entire colon of the rat and illustrate how this procedure permitted the quantitative assessment of aberrant crypt foci (ACF), a premalignant colon pathology, for characteristics consistent with progression to malignancy. ACF were detected by methylene blue staining and subjected to quantitative morphometric analysis. Colons were then restained with high iron diamine–alcian blue for assessment of mucin depletion using an image overlay to associate morphometric data with mucin depletion. The subsequent evaluation of ACF for beta-catenin staining is also demonstrated. The methods described are particularly relevant to the screening of compounds for cancer chemopreventive activity.

## 1 Introduction

Visualization of aberrant crypt foci (ACF) in methylene blue-stained colon whole mounts was first described by Bird [1] and gave insight into early pathologic changes in colon epithelium associated with carcinogen treatment and the subsequent development of colon cancer. Morphologic criteria used to identify ACF include: darkly stained epithelium, slight elevation of epithelial cells above the surrounding normal mucosa, increased pericryptal zone, enlarged crypt size, and changes in crypt shape [1-4]. While numerous ACF can be found within colons of carcinogen-treated animals, tumor multiplicity is quite low with one or two carcinomas observed per animal [5]. This situation has given rise to a controversy regarding the clinical relevance of ACF [3,6,7] since they have been widely used as a surrogate endpoint for colon carcinogenesis to screen compounds for cancer chemopreventive activity in preclinical models [8]. Loss of mucin occurs in a small percentage of ACF, and these mucin-depleted foci (MDF) have been proposed to identify ACF with an increased probability of progressing to cancer, but this concept also has come under scrutiny [5,9-12]. Herein, we describe methods and evaluate the usefulness of morphometric image analysis to yield insight into this problem.

Current approaches used to prepare and evaluate colon whole mounts for identification and characterization of ACF [1,12-15] have a number of issues, which are not conducive to consistent acquisition of digital images and subsequent image analysis. They include: (1) tissues mounted on filter paper or cork boards to flatten during fixation must be removed from the object for transillumination and visualization of the stained mucosa [1,16]; (2) high-quality image capture of free-floating stained specimens can be difficult or unfeasible due to specimen movement in fluid media and natural topographical changes present in the colonic tissue; (3) real-time ACF counting and scoring, using a dissecting microscope, are prone to errors from observer fatigue; (4) labeling and storage of free-floating tissue is problematic; and (5) comparative analysis of additional staining techniques is hindered by lack of consistent specimen orientation. These issues have impeded the development of a more complete understanding of ACF as a surrogate biomarker for colon cancer and may account, in part, for the apparent disparities that have been reported between the prevalence of ACF and subsequent development of colon carcinomas [5].

Based on our extensive work in developing techniques appropriate for preparing mammary gland whole mounts [17-19] and carrying out image analysis on digitally captured images of mammary tissue [20,21], we postulated that whole mounts of colon could be prepared by directly adhering the tissue to glass microscope slides and the whole mounted tissue then be used for semi-automated acquisition of contiguous images of the entire colonic mucosa; this would circumvent many of the limitations listed above. Moreover, we undertook the development of approaches to permit methylene blue staining, image acquisition, re-staining with high iron diamine–alcian blue (HID-AB) and further image acquisition in order to address current questions about the relevance of ACF detected by methylene blue versus those in which mucin depletion can be detected as predictors of subsequent development of colon carcinomas [5,9,22-24]. For this purpose, methods were developed for the construction of non-destructive overlay images to permit sequential evaluation of ACF, detected in methylene blue images, for mucin depletion. Additional techniques for processing of whole mount preparations, paraffin-embedding, and microtomy were developed so that ACF and mucin-depleted foci could be further interrogated for the presence of dysplasia and for beta-catenin accumulation by immunohistochemical analysis, since beta-catenin accumulation is directly linked to colon cancer development [10,23-25]. We report, in detail, the methods developed for these procedures and illustrate their use in the morphometric analysis of ACF.

## 2 Materials and Methods

Female Sprague Dawley rats were obtained from Taconic farms (Germantown, NY) at 20 days of age. At 21 days of age, rats were given an ip injection of 50 mg 1-methyl-1-nitrosourea/kg body weight (Ash Stevens, Detroit, MI) [26]. Rats were group-housed in an environmentally controlled room maintained at 22°C and 50% relative humidity with a 12 h light–dark cycle. They were fed a modification of the AIN-93G diet formulation [27] and deionized water ad libitum. The study was terminated 9 weeks following carcinogen injection. The work followed ethical guidelines approved by the Colorado State University Animal Care and Use Committee.

### 2.1 Whole Mount Preparation

At necropsy, rats were skinned, and a midline incision was made in the lower abdominal wall to expose the internal organs. A second incision was made at the pubic symphysis, and the pelvic bones were spread apart to reveal organs at the base of the abdomen. Reproductive organs were removed in toto and the gastrointestinal tract exposed. The colon was cut at the anus, carefully lifted out of the abdomen and excised above the ileo-cecal valve to visually maintain the anatomical orientation of the tissue. Colon length was measured in millimeters from the anus to the cecum, and residual colonic mesentery was removed using Adson forceps (George Tiemann & Co, Hauppauge, NY, Cat. No. 105-235-1). The cecum, anus, and rectum were removed, and the colon was trisected starting at the distal end into three equal lengthwise segments that were operationally defined as representing descending, transverse, and ascending regions, respectively. The colon was briefly immersed in phosphate-buffered saline (PBS) pH 7.4, and hardened fecal pellets were removed using a gentle milking action of the colonic tissue between the index finger and thumb. A longitudinal incision running the entire length of the colon was made using a small pair of scissors (George Tiemann & Co, Cat. No. 105-411), which had been modified in house using a bench grinder to blunt and smooth both tips in order to prevent perforation of the colon and minimize disruption of the fragile colonic mucosa while making the incision. Large deposits of soft fecal material were removed using Adson forceps, and the tissue was washed in PBS to remove residual fecal material from the colonic mucosa.

The colon was laid serosa side down onto a Kimwipe^®^ to wick away excess moisture. This process was repeated a few times by gently lifting the colon and placing it on a dry section of the Kimwipe^®^ until the serosal surface became tacky, i.e., increased resistance noted when separating the colon from the Kimwipe^®^. Whole mounts of colonic tissue segments were prepared by laying the serosal surface down onto 75 × 25 mm 3-aminopropyltriethoxysilane-treated microscope slides (Statlab Medical Products, McKinney, TX, Cat. No. 418). Adson forceps were used to carefully stretch the four corners of the tissue toward the edges of the slide. Once tacked down at the corners, Adson forceps were used to stretch the areas lying in between by carefully grasping a small portion of underlying serosa at the edge of the colon and slightly lifting the tissue off the glass while pulling toward the edge of the slide, being careful not to damage the delicate mucosa nor introduce air bubbles between the tissue and glass. This process was quickly repeated along all four sides until the tissue was stretched completely flat across the surface of the slide. Whole mounts were exposed to air for a period of 4 min prior to immersion in fixative to prevent tissue detachment from the glass slide. Whole mounts were fixed in 10% neutral buffered formalin for 24 h, rinsed in tap water for 15 min, and stored in 70% ethanol.

### 2.2 Methylene Blue Staining and Image Acquisition

**Figure 1:**
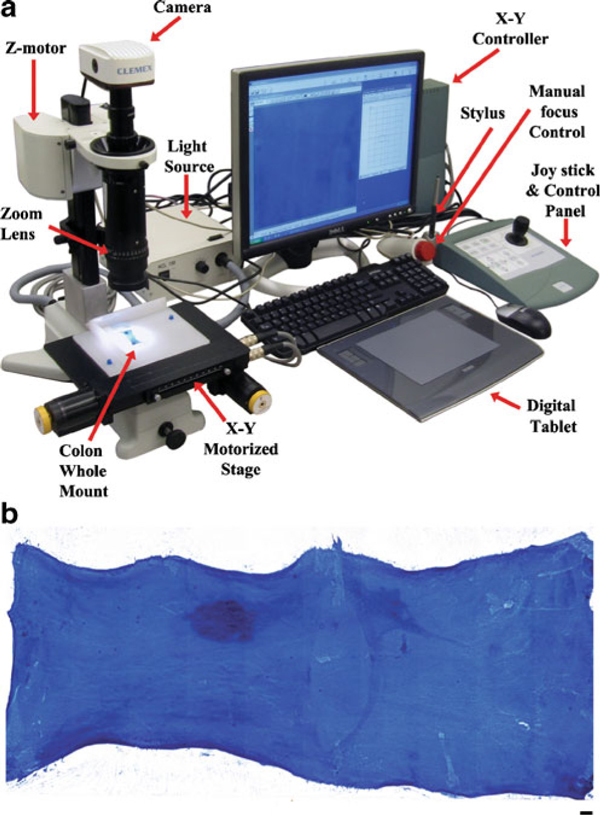
**a Workstation used to acquire high-resolution composite images of stained whole mount preparations**. **b** Colonic tissue whole mount on a glass microscope slide stained with methylene blue, bar = 1 mm.

Colon whole mounts were removed from 70% ethanol, rinsed in deionized water, and stained in 0.05% methylene blue (Sigma-Aldrich, St. Louis, MO., Cat No. M9140) for 3 min. Whole mount slides were rinsed in running deionized water until all excess methlyene blue stain had been removed. Digital images of stained colon whole mounts were captured using a customized image acquisition system (North Central Instruments, Inc., Plymouth, MN) consisting of a 3.0 megapixel CMOS digital camera (Clemex Technologies, Inc. Longueuil, Canada) mounted on a Z16 APO monocular zoom lens 16:1 with a magnification range of ×0.57–9.2 (Leica Microsystems, Inc., Bannockburn, IL). The camera and lens were mounted on a Z motor (Leica Microsystems, Inc.) attached to a transmitted light base with a 100 × 100 mm motorized stage (Clemex Technologies, Inc.). An X–Y control box and joystick (Clemex Technologies, Inc.) in conjunction with a Pentium 4 desktop PC (Dell, Round Rock, TX) and Captiva v4.0 software (Clemex Technologies, Inc.) were used for image acquisition (Figure 1a).

**Figure 2:**
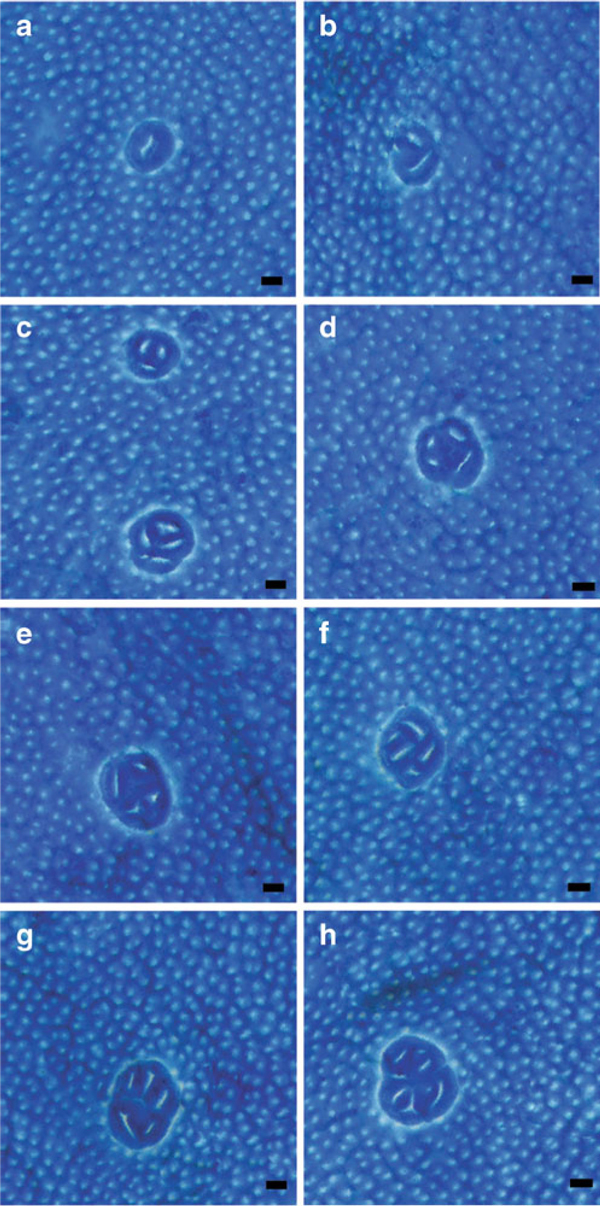
**a–h Examples of ACF in methylene blue-stained colon whole mount**. ACF are easily distinguished from surrounding normal colonic mucosa, bars = 100 μm.

Methylene blue-stained colon whole mounts were placed on a 6-mm thick sheet of white acrylic plastic (Gagne, Inc. Johnson City, NY) mounted on top of the motorized stage to act as a diffuser. Specimens were trans-illuminated using a 20 V/150 W halogen lamp light source (Volpi, Auburn, NY) with a daylight filter mounted at the rear of the base. A series of seamless tiled Z stack images (×1.6 objective) were captured automatically using the motorized stage in conjunction with the Captiva 4.0 software. The software automatically merged the tiled Z stack images together into a single uniformly focused composite image based on a best contrast algorithm (Figure 1b). Resulting images were saved as TIF files. High-resolution image acquisition enabled the user to easily identify ACF containing one or more crypts (Figure 2a–h).

### 2.3 Digital Extraction of ACF

Composite digital images were opened in Photoshop^®^ v9.0.1 (Adobe Systems, Inc., San Jose, CA) and saved as PSD files (Photoshop^®^ native format with support for layers), one file for each of the three whole mount slide preparations per animal representing ascending, transverse, and descending colon. ACF were individually circumscribed on a separate transparent layer using the Photoshop^®^ pencil tool with a contrasting bright color in conjunction with an Intuos 3 stylus and digital tablet (Wacom Technology Corp., Vancouver, WA). ACF were digitally excised from the image by selecting the transparent area outside the circumscribed borders using the magic wand tool and then applying the inverse command to select only the colored borders of the circumscribed ACF. The background layer was activated and that layer, via copy command, was used to create a new transparent layer containing only the excised ACF. The image was duplicated and flattened to remove all layers except the excised ACF on a white background. The resulting image was saved as a separate TIF file for image analysis.

### 2.4 HID-AB Staining

Methylene blue-stained colon whole mounts were stained with HID-AB as described by Caderni et al. [28] to look for evidence of mucin depletion in ACF. Images of HID-AB-stained colon whole mounts were acquired as outlined above and added to PSD files as a separate layer and aligned with the original methylene blue-stained images. Once aligned, the same layer used to circumscribe methylene blue-stained ACF was used to extract HID-AB-stained ACF as a separate layer using the technique described above, and the resulting image was saved as a separate TIF file for image analysis. The use of separate aligned layers permitted each image to be toggled on or off at will enabling qualitative assessment of the same ACF in both methylene blue and HID-AB stained whole mounts.

### 2.5 Image Analysis

**Figure 3:**
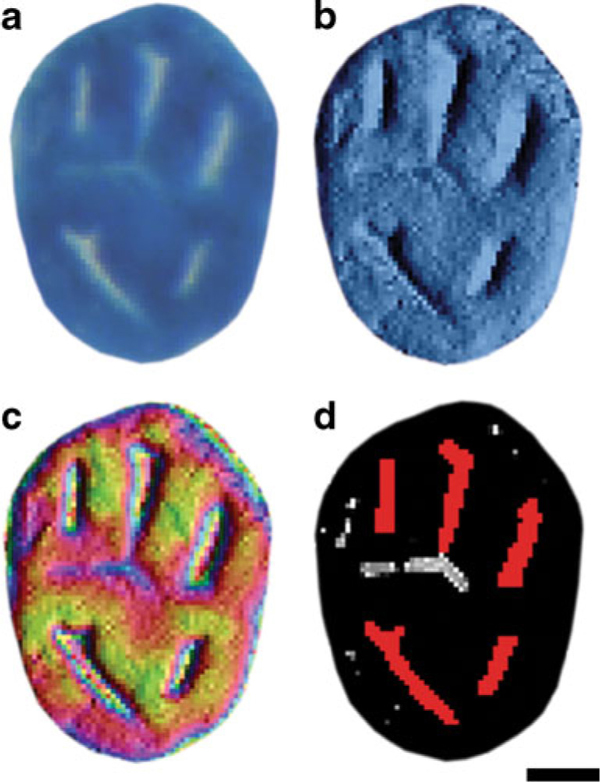
**a Methylene blue-stained ACF enlarged to show detail**. **b** Surface plot enhanced image of **a** showing ACF topography with crypt detail. **c** Surface plot image of **a** with *pseudo color* applied to show changes in both topography and density. **d** High-contrast image of **b** depicting software selected crypts in *red*. *Small gray*/*white regions* indicate areas lying below the crypt threshold, which were automatically discarded by the software. *Larger*, *elongated gray*/*white* indicate areas that were falsely identified as crypts by the software, but were removed by the user prior to analysis based on information gleaned from topographical views in **b** and **c**, i.e., slight invaginations were present on the surface of the ACF, but did not penetrate deep enough to be classified as a true crypt. Bar = 100 μm.

Separate macros were developed in Image-Pro Plus^®^ v4.5.0.27 (Media Cybernetics Inc., Bethesda, MD) to streamline analysis of ACF from both methylene blue and HID-AB-stained image files. In both macros, individual ACF were automatically selected for analysis from imported images. The methylene blue macro presented the user with multiple renderings of each ACF, which included the methylene blue-stained ACF image, a surface plot image depicting ACF topography with crypt detail, a pseudo-colored surface plot image showing changes in both topography and density and a high-contrast grayscale image with software identified crypt areas marked in red (Figure 3). The user was presented with options to toggle marked crypt areas on/off and split or join marked areas prior to analysis based on visual characteristics present in the other renderings. A number of morphometric parameters were measured, which included area, density, integrated optical density, maximum diameter, and roundness [(perimeter^2^)/(4*π *area)] of each ACF. The same parameters were applied to crypts lying within each ACF, and the total numbers of crypts per ACF were also measured.

The HID-AB macro used three different segmentation thresholds based on hue, saturation, and intensity to isolate areas within each ACF and place them into three separate classes based on color: HID (dark brown), AB (blue), and unstained (absence of brown or blue color). Class areas were measured and expressed as a percent of the total area for each ACF. Unstained areas, representing ≥85% of the total area of each ACF, were operationally defined as MDF. Morphometric data from both macros were exported via DDE to an Excel spreadsheet.

### 2.6 Whole Mount Tissue Processing, Paraffin-Embedding, and Microtomy

Colon whole mounts on glass slides were placed in Tissue Tek^®^ plastic slide racks (VWR, West Chester, PA, Cat. No. 25608-868) and processed in an automatic tissue processor using an abbreviated processing schedule and infiltrated with molten paraffin. Whole mounts were bisected down the long axis, and each half was trisected yielding six pieces of tissue per slide; tissues were embedded as separate blocks, mucosa side down. Five-micron serial sections were cut from each block, mounted onto 3-aminopropyltriethoxysilane-treated glass microscope slides, and stained with hematoxylin and eosin (H&E) according to normal laboratory protocol. Images of H&E sections were acquired as mentioned above and added to the PSD file as a separate layer. This layer was aligned with previously captured methylene blue and HID-AB layers, thus allowing qualitative assessment of ACF across all three staining techniques.

### 2.7 β-catenin Immunohistochemistry

Sections were cut at 5 μm and mounted on 3-aminopropyltriethoxysilane-treated slides and heat-immobilized in a 60°C oven for 20 min. Sections were deparaffinized in three changes of xylene, hydrated in a series of graded ethanols, rinsed in deionized water followed by three rinses in Tris-buffered saline (TBS) [50 mM Tris–HCl, 150 mM NaCl, pH 7.6 with 0.05% Tween 20 (Dako, Carpinteria, CA, Cat. No. S1968 and S1966)]. Subsequent steps were carried out at room temperature using an Autostainer (Dako, Carpenteria, CA). Anti-β-catenin (BD Biosciences, San Jose, CA, Cat. No. 610153) 1:50 was applied and incubated for 1 h followed by two rinses in TBS. FITC donkey anti-mouse Fab'2 (Jackson ImmunoResearch Laboratories, West Grove, PA, Cat. No. 715-096-151) 1:100 in 10% normal donkey serum (Jackson ImmunoResearch Laboratories, Cat. No. 017-000-121) was applied and incubated for 30 min followed by two rinses in TBS. 4',6-diamidino-2-phenylindole (DAPI) (Invitrogen, Carlsbad, CA, Cat. No. D1306) 300 nM was applied and incubated for 10 min followed by two rinses in TBS. Slides were rinsed in two changes of deionized water for 1 min and allowed to air dry under a fume hood in the dark.

Images were acquired using a Zeiss Axiocam HRm camera (Carl Zeiss, Thornwood, NY) coupled to a Zeiss Axioskope II microscope (Carl Zeiss). Multi-channel acquisition within the Axiovison v4.1 software (Carl Zeiss) was used to obtain separate images at a magnification ×400 for both FITC (filter excitation and emission of 480 and 535 nm, respectively) and DAPI (filter excitation and emission of 350 nm and 460 nm, respectively) stained areas, which were rendered as a composite image containing both fluorophores.

### 2.8 Statistical Analyses

Descriptive statistics, ANOVA, and multivariate analysis of variance were performed using SYSTAT v12.02 (SYSTAT Software, Inc., Chicago, IL).

## 3 Protocol

### 3.1 Necropsy

1. Excise the entire colon from cecum to anus as quickly as possible, i.e., <4 min from time of euthanasia.

2. Wet the serosal surface of the excised colon by quickly dipping in PBS pH 7.4

3. Remove hardened fecal pellets by gently milking the serosa between the index finger and thumb.

4. Make a longitudinal incision along the entire length of the colon using a pair of small, blunt-tipped scissors and remove any large deposits of soft fecal material.

5. Grasp the superior (proximal) end of the colon using a pair of Adson forceps (George Tiemann & Co, Hauppauge, NY, Cat. No. 105-235-1). Note the orientation of the mucosal surface and dip repeatedly in PBS until all visible fecal material is removed.

6. Remove the colon from PBS and lay the serosal side down on a Kimwipe^®^.

7. Trisect the colon into three equal length segments, representing ascending (proximal), transverse, and descending (distal) portions of the colon.

8. Place each segment on a separate Kimwipe^®^, maintaining orientation, and fold an unoccupied portion of the Kimwipe^®^ on top of the mucosal surface to wick away excess PBS.

9. Unfold the Kimwipe^®^, grasp the superior end of the colon, and gently peel off the Kimwipe^®^, and lay it down on a used portion of the paper. Repeat this process until all excess PBS has been wicked away and the serosa becomes tacky.

10. Use Adson forceps to carefully transfer the segment from the Kimwipe^®^ to a clean, 75 × 25-mm 3-aminopropyltriethoxysilane-treated microscope slide (Statlab Medical Products, McKinney, TX, Cat. No. 418), mucosal side up and orient the segment with the superior end toward the frosted label of the slide.

11. Use Adson forceps to gently grasp underneath the outer edge of the colon segment and pull toward the edge of the slide being careful not to disturb the fragile mucosa. Repeat this process all the way around the colon segment so that the serosal surface is mounted completely flat against the glass slide.

12. Allow the colon whole mount to air dry for 4 min to prevent detachment. Completely immerse the slide in 10% neutral buffered formalin and fix for 24 h. Rinse in tap water and store in 70% ethanol until ready to stain.

### 3.2 Methylene Blue Staining

1. Remove the slide(s) from 70% ethanol and rinse briefly in deionized water (dH_2_O) for 1 min. Do not stain more slides than can be imaged in a 2-h period.

2. Immerse the slide(s) in freshly prepared 0.05% methylene blue (Sigma, St. Louis, MO., Cat No. M9140) by dipping the slide 20× at 1-s intervals and then let sit undisturbed in stain for 3 min at room temperature (RT).

3. Rinse the slide(s) briefly in dH_2_O until all excess methlyene blue stain has been removed.

4. Remove one slide at a time from dH_2_O, capture images, and then store in 70% ethanol.

### 3.3 Circumscribing Methylene Blue-Stained ACF in Photoshop^®^

1. Open the captured image into Photoshop^®^, create a new layer, and label as "Outlines"

2. Click the Outlines layer to activate and use the Photoshop^®^ pencil tool (pixel diameter = 2, hardness = 100%) with a strong contrasting color, e.g., red to completely circumscribe each visible ACF. A stylus and digital tablet will increase the speed and efficiency of this process while greatly reducing fatigue associated with using a mouse.

3. Once all ACF have been circumscribed, use the Photoshop^®^ magic wand tool (tolerance = 30, continuous and anti-alias options checked), and click anywhere outside the circumscribed border of an ACF, i.e., in the blank portion of the Outlines layer.

4. Choose Select and Inverse from the Photoshop^®^ menu.

5. Click the background layer to make it the active layer. Place the cursor inside the circumscribed boundary of any ACF, right click, and choose Layer via Copy. This will create a new transparent layer with ACF only. Label the layer as "methylene blue ACF" and save the image file in .PSD format.

6. Turn off all layers with the exception of the methylene blue ACF layer and save a copy of the file in .TIF format (no layers). The image is ready for image analysis.

### 3.4 High Iron Diamine–Alcian Blue Staining

1. Remove the slide(s) from 70% ethanol and rinse in dH_2_O for 5 min.

2. Immerse the slides in a small staining dish of diamine solution consisting of 480 mg of *N*'-*N*'-dimethyl-m-phenylene diamine (Sigma, Cat. No. D3886), 80 mg of *N*'-*N*'-dimethyl-*p*-phenylene diamine (Sigma, Cat. No. D4139), 200 ml of H_2_O, and 5.6 mL of 60% ferric chloride (Sigma, Cat. No. F2877). Allow slides to stain for 18 h at RT.

3. Rinse the slides 3× in dH_2_O.

4. Immerse slides in a freshly prepared, filtered (No. 1 Whatman) solution of 1% Alcian blue (Sigma, Cat. No. A3157) in 3% acetic acid and stain for 30 min at RT.

5. Rinse slides 3× in 80% ethanol followed by 3× in dH_2_O.

6. Capture images and store in 70% ethanol.

### 3.5 Circumscribing HID-AB-Stained ACF in Photoshop^®^

1. Open the original methylene blue-stained .PSD image in Photoshop^®^.

2. Open the HID-AB-stained image, and drag the layer to the original methylene blue-stained image, which will create a new layer in the original image file. Label the layer as HID-AB, and arrange the layer in the palette such that it lies directly above the methylene blue-stained layer.

3. Reduce the opacity of the HID-AB layer from 100% to 50% in order to make the layer semi-transparent.

4. Click on Edit and Free Transform from the Photoshop^®^ menu and adjust the XY position of the HID-AB layer so that it is directly superimposed over the top of the methylene blue background layer, and all edges are aligned. Increase the opacity of the HID-AB to 100% once the layer is aligned.

5. Repeat steps 2–6 listed under method 3.3, "Circumscribing methylene blue-stained ACF in Photoshop^®^" (substituting "HID-AB ACF" for "methylene blue ACF") to create a new TIF image with only HID-AB-stained ACF. The image is ready for image analysis.

## 4 Results and Discussion

Advances in automated micro- and macroscopic image acquisition and digital image analysis have created the opportunity to investigate gastrointestinal disease processes, such as colon carcinogenesis and inflammatory bowel disease, with enhanced sensitivity and accuracy; however, a significant limitation in this field has been the limited application of quantitative morphometric approaches to the colon. Additionally, little detail has been reported in the literature on colon processing, and there is lack of uniformity in the methods that are described. To address these constraints, each step in the processing of the colon—excision, whole mount preparation, fixing, staining, processing, and histological or immunohistochemical evaluation—was evaluated and modified as appropriate. To address the issues related to this method's development, each phase is further discussed below.

### 4.1 Colectomy

Removal of the colon is associated with several problems which are mitigated by the following procedures. (1) The length of excised colon at the distal end can be influenced by variation among technicians in the excision process. Since a significant number of ACF are found in the descending colon [16,29-32], it is imperative that this portion of colonic tissue be removed fully intact. The incision through the pubic symphysis and clearing of reproductive organs accommodates full colon excision to the anus. (2) The forceps used to excise the colon from the abdomen can induce crush artifact, visible in methylene blue-stained whole mounts. Manual removal of the colon with the fingers minimizes the introduction of excision artifacts. (3) Excised colon segments can be difficult to maintain in the proper orientation (i.e., proximal vs. distal). Excising the colon with the cecum attached permits accurate anatomical orientation of the tissue for purposes of measurement and subsequent preparation of ascending, transverse, and descending colon segments on slides.

### 4.2 Whole Mount Preparation

To avoid tissue detachment from the slide, debris removal (e.g., mucus, mesentery, excess peritoneal fluid, and fecal material) is critical. By doing this, the requisite connective tissue is exposed providing improved spreading of the colon on the glass slide and better adhesion of the serosal surface to the slide. Washing the colonic tissue in PBS must be followed by blotting of the serosal surface to increase tackiness of the tissue. A brief period of exposure to ambient air prior to immersion in fixative also enhanced attachment of the colonic tissue to the glass. Significant amounts of gouging artifacts can result at the periphery of tissue when Adson forceps are employed for stretching. A modified technique utilizing forceps to anchor the corners first then stretching the areas lying in between is advisable to prevent damage to colonic tissue at the edge. The whole mount preparation is a fundamentally different approach to analysis of colonic ACF, compared with current protocols in the literature, making extensive staining and image analysis procedures possible.

### 4.3 Methylene Blue Staining

Protocols for methylene blue staining to detect ACF vary by diluent (water or saline), methylene blue concentration, staining time, and agitation technique during staining. Various combinations of these approaches were evaluated for the staining of the glass whole mounted colons and marked variations in staining intensity were noted. It was found that staining protocols in which buffered saline was used as the diluent, instead of deionized water, decreased staining affinity, and thus, higher concentrations of methylene blue stain and prolonged staining times (30–60 min) were necessary in order to achieve ACF visualization. Whole mounts stained with this method resulted in ACF that appeared muted and were difficult to distinguish from the normal mucosa. The methylene blue stain in unbuffered deionized water required lower concentrations of methylene blue stain and a shorter incubation period; this approach resulted in vibrantly stained ACF that were discernable from the normal mucosa (Figure 2a–h). Vertical agitation of slides in methylene blue stain was required to achieve uniform staining. Optimal staining was observed when whole mount slide racks containing evenly spaced slides were fully immersed and withdrawn from freshly prepared methylene blue stain, 20 times at 1-s intervals and then allowed to sit undisturbed in methylene blue for 3 min. Attempts were made to mimic manual agitation using various mechanical devices, e.g., magnetic stir bar in a staining dish, an orbital shaker, etc. However, all of the mechanical devices evaluated proved inferior to the manual staining technique.

### 4.4 Image Acquisition

**Figure 4:**
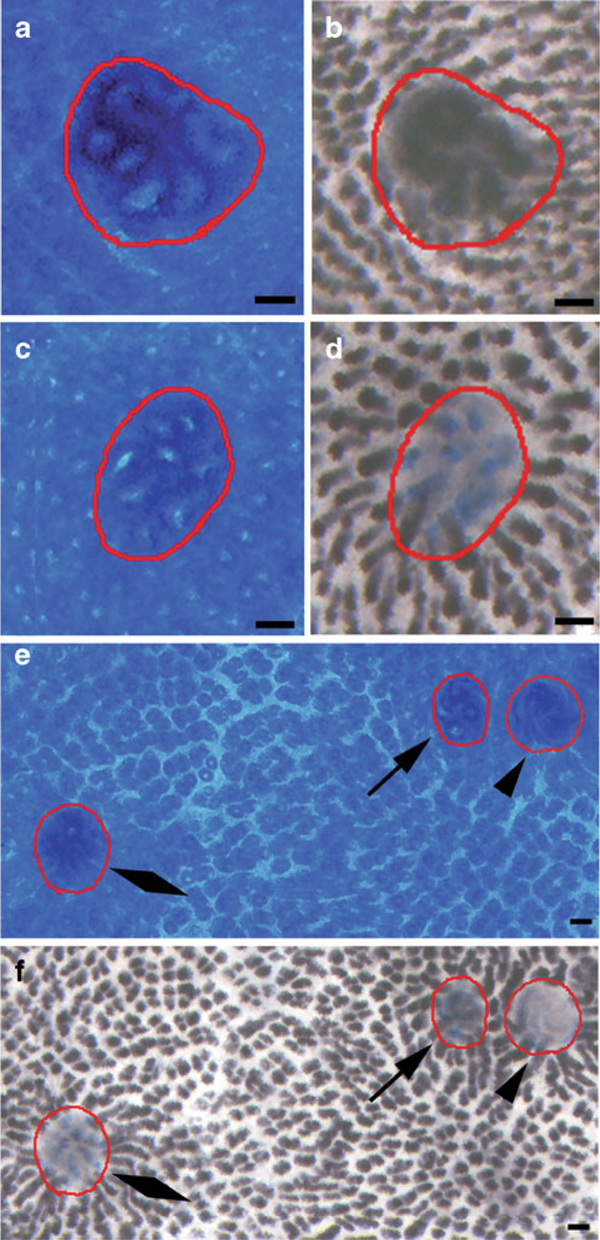
**Methylene blue-stained ACF (a, c) and corresponding HID-AB stain (b, d)**. ACF with enlarged round shaped crypts (**a**, **b**) appeared to have increased deposition of HID or more sulphomucin whereas ACF with slit-like crypts (**c**, **d**) demonstrated little to no HID staining with variable amounts of AB stain, i.e., sulphomucin-depleted. **e** Methylene blue-stained colon with three ACF showing variable crypt morphology. *Round* crypts (*arrow*), *slit*-*like* crypts (*diamond*) and *spiral* crypts (*arrowhead*). **f** Corresponding HID-AB-stained colon. *Round* crypts favored both HID and AB stains (*arrow*). *Slit*-*like* crypts typically demonstrated AB only with scant HID stain (*diamond*). *Spiral* crypts showed absence of HID and scant AB stain. Bars = 100 μm.

Though initially based on manual capture and tiling of images with a digital camera, macro zoom lens, and Photoshop^®^ software, an integrated approach to acquire a contiguous series of tiled images at various focal planes was adopted. Forming a single-focus composite rendered image of the entire whole mount slide utilizing the motorized stage provided an effective solution for problems associated with manual acquisition. Manual capture and tiling resulted in loss of process control, particularly image quality, which obfuscated ACF crypt number determination by image analysis software. Hence, the semi-automated approach is considered essential for reducing observer fatigue-dependent errors and increasing the accuracy and reproducibility associated with quantification of the number of ACF and the number of crypts within an ACF. In addition, automated image capture and tiling improved control over image quality, thereby creating the opportunity for non-destructive overlays of multiple differential stains applied to the same sample (Figure 4).

### 4.5 High Iron Diamine–Alcian Blue Stain

**Figure 5:**
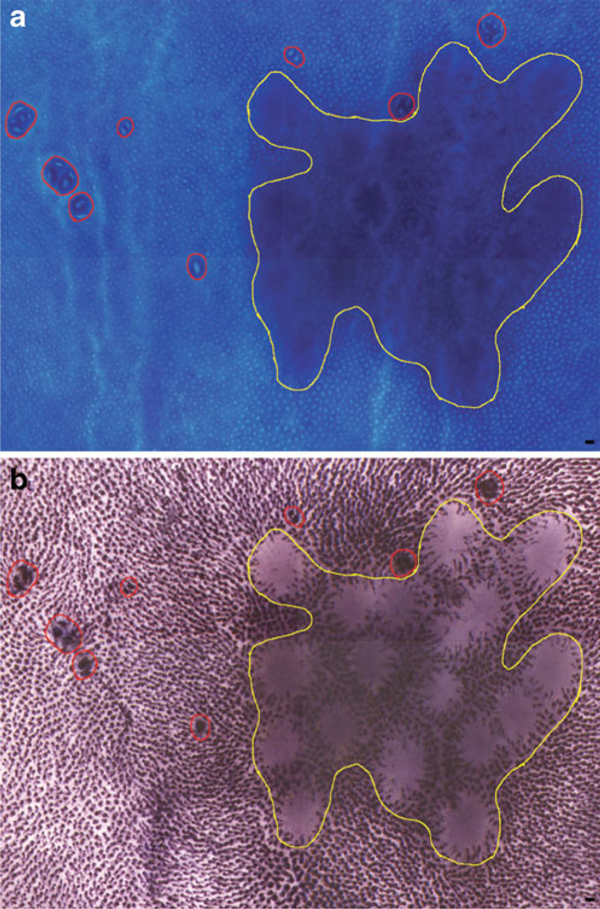
**a Methylene blue-stained colon whole mount with several ACF circumscribed in *red* and GALT circumscribed in *yellow***. **b** HID-AB stain applied to the same whole mount. Several unstained areas appear within the GALT, but are not true MDF or ACF.

Image acquisition of HID-AB-stained whole mounts required increased camera exposure times due to the darkly stained crypts of the normal mucosa. ACF were measured for percent stained area of HID-AB and placed into four operationally defined categories based on stain deposition: abundant sulphomucin in the form of HID accumulation (dark brown color only); mixed sulpho- and sialomucin (brown and blue colors); sulphomucin depletion represented by absence of HID staining with AB stain (blue color only); sulpho- and sialomucin depletion represented by lack of either HID or AB staining (no color; Figure 4). This level of characterization should facilitate an increased understanding of the relationship of mucin secretion and depletion to other pathogenic events in the colon. Areas lying within gut-associated lymphoid tissue (GALT) may be devoid of stain when evaluating AB or HID-AB-stained whole mounts, thus masquerading as MDF (Figure 5). The ability to toggle stain image layers on and off within Photoshop^®^ proved vital in locating GALT, which was easily indentified in the methylene blue-stained layer. While no ACF were identified within GALT, there were instances where ACF appeared at the periphery of GALT (Figure 5).

### 4.6 Image Analysis

The low degree of contrast between ACF and surrounding normal mucosa in methylene blue-stained whole mounts presented a significant challenge for automated image analysis. The authors are unaware of any software algorithm to date that consistently and reliably identifies ACF in methylene blue-stained colon whole mounts. Though this technological advance would likely shorten analysis time and improve reproducibility, the lack of such an approach necessitated direct user interaction. As opposed to the constraints placed on users of Image-Pro Plus^®^ software, Adobe Photoshop^®^ enabled ACF to easily be circumscribed using a non-destructive layer and improved work flow control. Use of Photoshop^®^ was also key to developing overlay capability and studying the staining congruence and divergence of histological features from a single ACF. Multiple renderings of each ACF image were used to facilitate accurate selection of crypts prior to analysis. While many crypts are easily identified, there are instances where slight invaginations can masquerade as crypts. Use of surface plot enhanced images enabled greater definition of the two-dimensional image, which allowed the observer to discard false-positives prior to analysis (Figure 3).

### 4.7 Whole Mount Processing and Paraffin-Embedding

Obtaining flat tissue sections for embedding and microtomy was complicated by the tendency for the thin tissue to curl. The approach that we adopted to overcome this problem involved direct processing of the whole mounts while attached to glass slides, providing for adequate reagent transfer into the tissue and keeping it flat. An abbreviated processing schedule was used to decrease the amount of dehydration dependent shrinkage artifact. Tissue processed in this manner remained attached to the glass slide through all processing stages including paraffin infiltration. Attempts to use disposable plastic base molds proved ineffective while stainless steel base molds produced consistently flat paraffin blocks.

### 4.8 Microtomy and Staining

**Figure 6:**
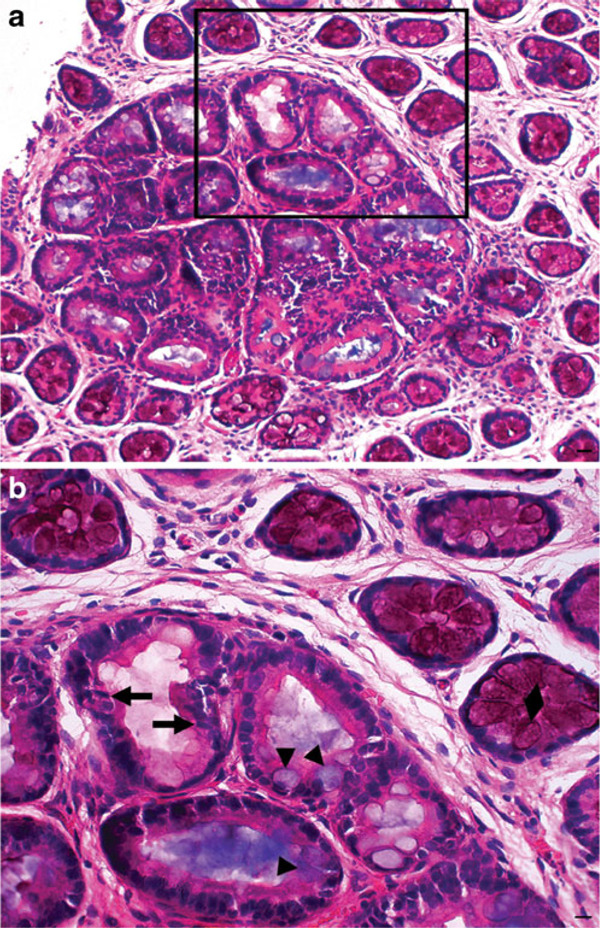
**a H&E stain showing distinct ACF from surrounding normal crypts (×100); bar = 100 μm**. **b** ACF at higher magnification (×400) with *arrows* indicating cigar shaped dysplastic nuclei. HID staining (sulfomucin) is evident in the goblet cells of surrounding normal crypts (*diamond*), but is absent in particular ACF. AB staining (sialomucin) can be seen in the goblet cells (*arrowheads*) within the ACF. However, the dysplastic crypt shows absence of staining for both mucins; bar = 50 μm.

Proper alignment of the rotary microtome object holder with respect to the block minimized excessive facing of the tissue, and blocks embedded with the mucosal surface oriented down were efficiently cut and produced more uniform paraffin ribbons. Obtaining a single, complete representative 5-μm section of each block was difficult and therefore two to three serial sections were used to reconstruct full cross-sections. Attempts to excise individual ACF from whole mount tissue after HID-AB staining, using a dissecting microscope and a small steel punch, proved tedious, even though it produced classic longitudinal sections with representation from all layers of the colonic tissue. Cross-sections of colon whole mounts were easier to produce and had the unique advantage of being imaged and overlaid onto previously captured methylene blue and HID-AB stained images. H&E-stained ACF prepared in this manner was easily distinguished from the surrounding mucosa (Figure 6).

### 4.9 Morphometric Analysis of ACF

Colons from 21 rats were subjected to morphometric analysis. The incidence of ACF identified by methylene blue staining in these carcinogen-treated animals was 100% with 73 ± 27 ACF/rat (mean ± SD). No ACF was found in saline-treated controls (data not shown). Subsequent staining of the same ACF with HID-AB indicated that 13 of the rats had a least one MDF with MDF-bearing rats having 3 ± 2 MDF/rat (mean ± SD). When the colons of the rats were excised, they were trisected into the ascending, transverse and descending segments as described in the Materials and Methods section permitting the analysis of ACF (non-MDF and MDF) by colon segment (Table 1). The transverse region of the colon was found to contain the highest number of ACF, while the descending region contained the highest number of MDF; the ascending region had the fewest number of ACF and MDF. These findings are consistent with those reported previously [10]. We also computed an index of MDF divided by ACF and found that it increased across colon segments (ascending<transverse<descending colon; 0.9%, 2.4%, 4.0%, respectively, *p* = 0.04). Whether such an index would have value in chemoprevention research is a concept that could easily be evaluated using the methods described herein with the goal of resolving current disagreements about the relative value of ACF versus MDF in identifying agents that have the potential to inhibit the development of colon cancer [3,6,7,33].

**Table 1:** Incidence, total, and average number of ACF (non-MDF and MDF) by colonic region

		Ascending	Transverse	Descending
Incidence (%)	Non-MDF	85.7	100	100
	MDF	9.5	38.1	42.9
Total number	Non-MDF	202	800	549
	MDF	2	19	22
Average number	Non-MDF	9.6 ± 10.6	38.1 ± 14.7	26.1 ± 12.1
	MDF	0.1 ± 0.3	0.9 ± 2.0	1.1 ± 1.5

Mean colon length (millimeters) = 150.5 ± 2.3, (mean ± SEM), *n* = 21 rats.

Values are means ± SD

The methods described are performed serially on the colon, but the digital acquisition of images and the ability to overlay multiple images permits parallel analysis of colon crypts in a manner not previously reported. To illustrate this capability, methylene blue-identified ACF were evaluated for morphometric criteria (Table 2) based on the presence or absence of mucin depletion (see Supplementary Materials for additional morphometric data). Using this approach, several morphometric measurements were found to be statistically different (*p* ≤ 0.05) between MDF and non-MDF when observing ACF containing ≥5 crypts (Table 3), an ACF crypt count number generally associated with greater risk for colon cancer [12]. Despite the finding of statistical differences in a subset of morphometric measurements, it was notable: (1) that 30% of MDF occurred in ACF with less than five crypts per focus, (2) that the values for the significant morphometric measurements that distinguished between MDF-positive and MDF-negative ACF revealed that MDF were smaller and less intensely stained by methylene blue (Table 3), and (3) that we could not identify a set of morphometric measurements of ACF that predicted which ACF would be mucin-depleted. These findings demonstrate the power of the approach and indicate that application of this method may provide data to clarify the current controversy about the relative value of ACF versus MDF as surrogate biomarkers for colon cancer.

**Table 2:** Morphometric measurements for which aberrant crypts were evaluated

Measurement type	Measurement name	Description with units	Average value(mean±SD)
Classification	CRYPTCOUNT	Total number of crypts per ACF	6.7 ± 5.4
Area	ACF_AREA	Area of each ACF in square microns (μm^2^ × 10^4^)	2.5 ± 1.5
	ACF_EPIAREA	ACF epithelial area (μm^2^ × 10^4^) (ACFAREA–CSUMAREA)	2.3 ± 1.3
	C_SUMAREA	Sum of crypt area (μm^2^ × 10^3^)	2.2 ± 2.5
	C_AVGAREA	Average crypt area in square microns (μm^2^ × 10^2^)	3.4 ± 1.7
Diameter	ACF_DIAAVG	ACF average diameter in microns (μm × 10^2^)	1.7 ± 0.5
	C_AVGMAXDIA	Crypt maximum diameter in microns (μm × 10)	3.0 ± 1.0
	C_AVGSIZELEN	Crypt average size length in microns (μm × 10)	2.9 ± 1.1
Perimeter	ACF_PERIM	ACF perimeter in microns (μm × 10^2^)	5.6 ± 1.6
	C_AVGPERIM	Average crypt perimeter in microns (μm × 10)	6.8 ± 2.7
Roundness	ACF_ROUND	ACF roundness (1 = round, higher numbers indicate polygonal shape)	1.1 ± 0.1
	C_AVGROUND	Average crypt roundness (1 = round, high numbers indicate polygonal shape)	2.2 ± 0.6
	C_ROUNDCAT	Crypt roundness category: 0–4	1.7 ± 0.7
Density	ACF_DEN	ACF density	0.5 ± 0.2
	ACF_IOD	ACF integrated optical density (area × density) (μm^2^ × 10^4^)	1.2 ± 0.9
HID-AB staining	P_SULFOAREA	Percent sulfomucin area (HID stain only) (%)	55 ± 32
	P_SIALOAREA	Percent sialomucin area (AB stain only) (%)	16 ± 20
	P_UAREA	Percent unstained area (lacks sulfo- and sialomucin HID-AB stain; high numbers, e.g., ≥85% probably indicate MDF) (%)	30 ± 26
	MDF	Mucin-depleted foci (≥85% PUAREA)	**–**

**Table 3:** Morphometric measurements of ACF with crypts ≥5 that differentiate non-MDF and MDF

ACF	**Area (μm**^**2**^**)**	IOD	Diameter (μm)	Perimeter (μm)
Non-MDF (*n* = 819)	32,207 ± 522	15,134 ± 336	195 ± 2	650 ± 5
MDF (*n* = 28)	25,820 ± 1429	10,995 ± 839	176 ± 5	593 ± 16
*p*	0.02	0.02	0.03	0.04

Values are means ± SEM based on number of crypts ≥5 per ACF

Statistically significant differences were observed, *p* ≤ 0.05

### 4.10 β-catenin Immunohistochemistry

**Figure 7:**
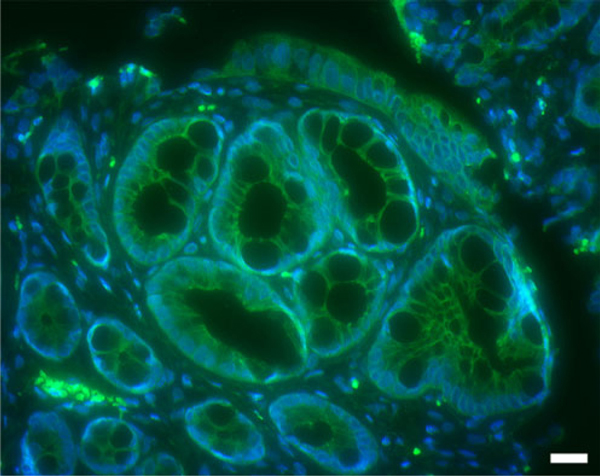
**Fluorescent immunohistochemical demonstration of β-catenin (FITC) in a 5-μm paraffin obtained from a colon whole mount previously stained with methylene blue**. An ACF is shown in the center of the image with smaller, normal crypts at the periphery. No evidence of β-catenin accumulation and/or nuclear translocation is noted in this example. Bar = 20 μm.

In view of the results of the morphometric analyses, a method was developed which allowed serial evaluation of β-catenin staining in 5-μm paraffin sections obtained from HID-AB-stained whole mounts as reported by Femia et al. [10]. However, the staining quality was far below that of typical sections yielding an overall muddy appearance that could not be evaluated; the reasons for our inability to reproduce results as reported in [10] are unclear. In contrast, sections from methylene blue-stained whole mounts yielded crisp staining characteristics (Figure 7). Consequently, we recommend that future work, initiated to resolve the current ACF-MDF controversy, be performed by first detecting β-catenin in excised ACF prior to staining for mucins with HID-AB. The goal would be to be to identify ACF characteristics that are associated with β-catenin accumulation and nuclear translocation and to juxtapose that information with crypt characteristics associated with mucin depletion.

### 4.11 Technical Effort

The procedures described permit the digital acquisition of sequential images of the colon that can be sequentially analyzed for morphometric data, and these high-quality images of the entire colon can be archived for future use. These procedures are routinely performed in our laboratory and are taught to individuals with either an associate's degree or a bachelor of science degree by an individual who is well-versed in the procedures. Classification of dysplasia and the diagnosis of cancer is the only aspect of these procedures that requires advanced training. Since rodent colons are processed and evaluated by a number of procedures in various laboratories and individuals differ in the efficiency with which they can complete tasks in their respective methods, a summary of the amount of time required for each major step in the procedures is outlined in order to provide a framework for evaluating the merits and limitations of adopting the process described herein (Table 4).

**Table 4:** Estimated procedure and training time

Phase	Procedure	**Procedure time (min)**^**a**^	**Training time (min)**^**b**^
Necropsy	Colon excision and cleaning	1	10
	Whole mount preparation	2	20
ACF identification and analysis	Methylene blue staining^c^	4	8
	Image acquisition	8	24
	Circumscribing ACF	15	60
	ACF image extraction	1	5
	Methylene blue image analysis	15	60
MDF identification and analysis	HID-AB staining^c^	5	10
	Image acquisition	8	0
	Image layer alignment	1	10
	MDF image extraction	1	0
	HID-AB image analysis	10	30
Total time		71	237

^a^Procedural time required per slide, three slides per animal comprising the ascending, transverse, and descending colon

^b^Recommended one-time training. Does not require an advanced level degree or certification

^c^Represents hands on time of multiple slides stained in batch rather than stained individually

## 5 Conclusions

The approaches detailed in this paper are currently being used to qualitatively and quantitatively compare carcinogen-induced premalignant colon pathologies based on morphological and biochemical characteristics of whole mount preparations. These methods, involving staining with methylene blue and HID-AB, followed by analysis of the same lesions using light microscopy and traditional histochemical and immunohistochemical techniques, should provide the following opportunities: (1) enhancement of mechanistic investigation into ACF molecular pathology and establishment of premalignant endpoints predictive of subsequent development of colon carcinomas; (2) the development of more robust screening approaches for preventive agents and dietary factors that impact colon carcinogenesis; and (3) improvement of the design of intervention studies in animal models, especially where changes in mucin metabolism occur during the disease process, such as inflammatory bowel diseases.

## Abbreviations

ACF: aberrant crypt foci; GALT: gut-associated lymphoid tissue; H&E: hematoxylin and eosin; HID-AB: high iron diamine–alcian blue; MDF: mucin-depleted foci; PBS: phosphate-buffered saline; TBS: Tris-buffered saline

## Supplementary Material

Additional file 1 Click here for file
