# Supplementary material for: A Method for Serial Tissue Processing and Parallel Analysis of Aberrant Crypt Morphology, Mucin Depletion, and Beta-Catenin Staining in an Experimental Model of Colon Carcinogenesis
Source: Biol Proced Online. 2010 May 18;12:118–30. doi: 10.1007/s12575-010-9032-x (PMC3284111; doi:10.1007/s12575-010-9032-x)
Supplement: Additional file 1 [file 1480-9222-12-1-9032-S1.doc]

**Electronic Supplementary Material – Biological Procedures Online**

“A Method for Serial Tissue Processing and Parallel Analysis of Aberrant Crypt Morphology, Mucin Depletion, and Beta-Catenin Staining in an Experimental Model of Colon Carcinogenesis”

John N. McGinley, Matthew D. Thompson and Henry J. Thompson

**Supplementary Figures:**

Figure S.1 Counts of ACF by CRYPTCOUNT

Figure S.2 Counts of ACF by CRYPTCOUNT grouped by MDF classification

Figure S.3 Area morphometric measurements by CRYPTCOUNT

Figure S.4 Diameter morphometric measurements by CRYPTCOUNT

Figure S.5 Perimeter morphometric measurements by CRYPTCOUNT

Figure S.6 Roundness morphometric measurements by CRYPTCOUNT

Figure S.7 Density morphometric measurements by CRYPTCOUNT

Figure S.8 Staining morphometric measurements by CRYPTCOUNT

**Notes:**

Figures S.3-S.8 display means with standard error bars. Only data for CRYPTCOUNT (1-20) where the number of crypts per ACF is ≥1 and ≤ 20 is presented. This is due to the limited number of ACF with the number of crypts being >20. The low number (N) made estimates of variance unstable in ACF > than 20 crypts.

See below for descriptions of each measurement with units (not shown in supplemental figures).

| **Measurement Type** | **Measurement Name** | **Description with Units** |
| --- | --- | --- |
| **Classification** | **CRYPTCOUNT** | Total number of crypts per ACF |
| **Area** | **ACF_AREA** | Area of each ACF in square microns (µm^2^) |
|  | **ACF_EPIAREA** | ACF Epithelial Area (ACFAREA – CSUMAREA) (µm^2^) |
|  | **C_SUMAREA** | Crypt Sum Area (µm^2^) |
|  | **C_AVGAREA** | Crypt average area in square microns (µm^2^) |
| **Diameter** | **ACF_DIAAVG** | ACF Average Diameter in microns (µm) |
|  | **C_AVGMAXDIA** | Crypt Maximum Diameter in microns (µm) |
|  | **C_AVGSIZELEN** | Crypt Average Size Length in microns (µm) |
| **Perimeter** | **ACF_PERIM** | ACF Perimeter in microns (µm) |
|  | **C_AVGPERIM** | Crypt average perimeter in microns (µm) |
| **Roundness** | **ACF_ROUND** | ACF roundness (1 = round, higher numbers indicate polygonal shape) |
|  | **C_AVGROUND** | Crypt average roundness (1 = round, high numbers indicate polygonal shape) |
|  | **C_ROUNDCAT** | Crypt Roundness Category: 0-4 |
| **Density** | **ACF_DEN** | ACF density |
|  | **ACF_IOD** | ACF Integrated Optical Density (area x density) (µm^2^) |
| **Staining** | **P_SULFOAREA** | Percent Sulfomucin Area (HID stain only) (%) |
|  | **P_SIALOAREA** | Percent Sialomucin Area (AB stain only) (%) |
|  | **P_UAREA** | Percent Unstained Area (lacks sulfo and sialo mucin HID-AB stain; high numbers, e.g. ≥ 85 % probably indicate MDF) (%) |
|  | **MDF** | Mucin Depleted Foci, (>= 85% PUAREA) |

**Figure S.1 Counts of ACF by CRYPTCOUNT**

**Figure S.2 Counts of ACF by CRYPTCOUNT grouped by MDF classification**

**Figure S.3 Area morphometric measurements by CRYPTCOUNT**

**Figure S.4 Diameter morphometric measurements by CRYPTCOUNT**

**Figure S.5 Perimeter morphometric measurements by CRYPTCOUNT**

**Figure S.6 Roundness morphometric measurements by CRYPTCOUNT**

**Figure S.7 Density morphometric measurements by CRYPTCOUNT**

**Figure S.8 Staining morphometric measurements by CRYPTCOUNT**
